# Multi-objective evolutionary algorithms for analysis of conductance correlations involved in recovery of bursting after neuromodulator deprivation in lobster stomatogastric neuron models

**DOI:** 10.1186/1471-2202-14-S1-P370

**Published:** 2013-07-08

**Authors:** Atish Malik, Kenneth Shim, Astrid A Prinz, Tomasz G Smolinski

**Affiliations:** 1 Department of Computer and Information Sciences, Delaware State University, Dover, DE 19901, USA; 2 Department of Biology, Emory University, Atlanta, GA 30322, USA

## 

Neurons in the crustacean stomatogastric ganglion (STG) receive neuromodulatory inputs from higher centers through the stomatogastric nerve (stn). After the stn is cut or blocked, the stereotypical bursting activity of the system ceases, as STG neurons initially lose their activity pattern. Interestingly, however, the neurons typically recover their function within 24 to 96 hours and again exhibit activity similar to that in intact STGs [1]. This phenomenon is seen across different species (*e.g*., lobsters, crabs, *etc*.) and various neuron types (*e.g*., intrinsically bursting and spiking neurons). One possible explanation for its occurrence is coregulation of ionic current levels, and specifically the changes that appear to take place in such relationships in response to deafferentation (*i.e*., neuromodulator deprivation) [2]. Although the interaction between deafferentation, function recovery, and coregulation of ionic currents is under active research, the underlying mechanisms are not well understood. Here, we propose a computational approach to study these phenomena in two very important STG neurons: the anterior burster (AB) and pyloric dilator (PD), which together form the pacemaking kernel in the pyloric central pattern generator (CPG), which drives the tri-phasic rhythmic activity of the pyloric network. As the starting point in our study, we use the hand-tuned AB and PD Hodgkin-Huxley-type conductance-based models proposed in [3]. We define a parameter search space centered around those models by extending the ranges of the values for each of the parameters (12 for AB and 11 for PD) to -100% and +400% of the original hand-tuned values. We then utilize multi-objective evolutionary algorithms (MOEA) to explore the parameter space in search of models that exhibit electrical activity resembling that of neurons in presence of neuromodulation, despite being simulated without it (which is achieved by the removal of the modulatory proctolin current in the AB model, and a decrease in the maximum membrane conductance of the calcium currents in the PD model, as described in [3]). Specifically, we look at the period, burst duration, spike and slow wave amplitude, number of spikes per burst, spike frequency, and after-hyperpolarization potential, *etc*., which all constitute separate objectives in the MOEA, and must be within limits determined in physiological experiments for a model to be deemed acceptable. We consider such models to represent "recovered" neurons, as they function despite neuromodulation deprivation [4]. We then explore the model parameter search space for relationships between the parameter values (*i.e*., maximum membrane conductances) for the AB and PD neurons in isolation, as well as the entire pacemaker.
